# Nanosecond PEF Induces Oxidative Stress and Apoptosis via Proteasomal Activity Inhibition in Gastric Adenocarcinoma Cells with Drug Resistance

**DOI:** 10.3390/ijms232112943

**Published:** 2022-10-26

**Authors:** Julita Kulbacka, Nina Rembiałkowska, Anna Szewczyk, Joanna Rossowska, Małgorzata Drąg-Zalesińska, Marek Kulbacki, Anna Choromańska

**Affiliations:** 1 Department of Molecular and Cellular Biology, Faculty of Pharmacy, Wroclaw Medical University, Borowska 211 A, 50-556 Wroclaw, Poland; 2 Department of Animal Developmental Biology, Faculty of Biological Sciences, University of Wroclaw, 50-335 Wroclaw, Poland; 3 Hirszfeld Institute of Immunology and Experimental Therapy, Polish Academy of Sciences, 50-422 Wroclaw, Poland; 4 Division of Histology and Embryology, Division of Human Morpholog and Embryology, Faculty of Medicine, Wroclaw Medical University, 50-368 Wroclaw, Poland; 5 Polish-Japanese Academy of Information Technology, 02-008 Warsaw, Poland; 6 DIVE IN AI, 53-307 Wroclaw, Poland

**Keywords:** gastric adenocarcinoma, drug resistance, nsPEF, calcium ions, apoptosis, oxidative stress, proteasomal activity

## Abstract

Nanosecond (ns) pulsed electric field (PEF) is a technology in which the application of ultra-short electrical pulses can be used to disrupt the barrier function of cell plasma and internal membranes. Disruptions of the membrane integrity cause a substantial imbalance in cell homeostasis in which oxidative stress is a principal component. In the present study, nsPEF-induced oxidative stress was investigated in two gastric adenocarcinoma cell lines (EPG85-257P and EPG85-257RDB) which differ by their sensitivity to daunorubicin. Cells were exposed to 200 pulses of 10 ns duration, with the amplitude and pulse repetition frequency at 1 kHz, with electric field intensity varying from 12.5 to 50 kV/cm. The electroporation buffer contained either 1 mM or 2 mM calcium chloride. CellMask DeepRed visualized cell plasma permeabilization, Fluo-4 was used to visualize internal calcium ions content, and F-actin was labeled with AlexaFluor^®^488 for the cytoskeleton. The cellular viability was determined by MTT assay. An alkaline and neutral comet assay was employed to detect apoptotic and necrotic cell death. The luminescent method estimated the modifications in GSSG/GSH redox potential and the imbalance of proteasomal activity (chymotrypsin-, trypsin- and caspase-like). The reactive oxygen species (ROS) level was measured by flow cytometry using dihydroethidium (DHE) dye. Morphological visualization indicated cell shrinkage, affected cell membranes (characteristic bubbles and changed cell shape), and the reorganization of actin fibers with sites of its dense concentration; the effect was more intense with the increasing electric field strength. The most significant decrease in cell viability and GSSG/GSH redox potential was noted at the highest amplitude of 50 kV/cm, and calcium ions amplified this effect. nsPEF, particularly with calcium ions, inhibited proteasomal activities, resulting in increased protein degradation. nsPEF increased the percentage of apoptotic cells and ROS levels. The EPG85-257 RDB cell line, which is resistant to standard chemotherapy, was more sensitive to applied nsPEF protocols. The applied nsPEF method disrupted the metabolism of cancer cells and induced apoptotic cell death. The nsPEF ability to cause apoptosis, oxidative stress, and protein degradation make the nsPEF methodology a suitable alternative to current anticancer pharmacological methods.

## 1. Introduction

Electroporation is still a developing technique and finds usability mainly for drug or gene delivery or in food technology [1,2]. Nanosecond electric pulsed fields (nsPEFs) are mainly applied in food technology known as bio-based industry [3,4], but researchers still demonstrate numerous nsPEFs’ advantages in anticancer protocols [5,6,7,8]. The nanosecond range of the electric pulses induces various changes in the exposed cells. The main advantage is the high efficacy of permeabilization levels of inner and outer cell membranes [9]. This phenomenon provokes lipid membrane unsealing, enabling drug delivery [10,11], and then runs the processes leading to the alternation of cell metabolism and finally cell death [12,13]. Drug delivery seems a promising application in the case of anticancer protocols, in particular with drug resistance. NsPEFs enable the effective transport of impermeable molecules through outer and inner cell membranes, which causes more alternations in cells than conventional milli- or microsecond electroporation [14].It was also demonstrated that nsPEFs exposure causes an uncontrolled increase in calcium concentration due to the formation of membrane nanopores [15]. This phenomenon was used for the increased influx of drug molecules. Our previous study revealed that ultrashort electric pulses could be used for the increased delivery of Photofrin^®^ II in photodynamic therapy [10], strontium ranelate [16], and doxorubicin [17], bleomycin [6], and calcium ions [18]. The available studies also indicate promising anticancer effects in the case of a combination with cisplatin [7], gemcitabine [19], or adriamycin [20]. This study aimed to use low concentrations of calcium chloride with nsPEFs against human gastric cancer cells with drug resistance. Up to now, electroporation-based methods have had limited applications in gastric cancer therapy. Clinical trials for non-curable gastric cancer were undertaken by the Zealand University Hospital (Denmark) [21], where electrochemotherapy (ECT) with bleomycin was used. Klein et al. applied irreversible electroporation (IRE), and ECT—IRECT with bleomycin in patients with lymph node metastases from gastric cancer [22]. In another study, ECT was also used efficiently in treating peristomal tumors [23]. Kambe et al. used cisplatin and 5-fluorouracil to treat two gastric cancer cell lines: GCIY and KATO III [24]. The available in vitro studies highlight nanosecond electric pulses as an effective method in the case of cancers resistant to chemotherapy. It was recently shown that micro- and nanosecond electroporation enhanced the cytotoxic effect of doxorubicin [17] or calcium ions [18] in drug-resistant breast and colon cancer cells. Calcium homeostasis is one of the most important physiological factors for each cell [25]. Any disturbance of this homeostasis, whether by g. calcium overload or the lack of it, disrupts the functioning of the cell and directs it to the apoptosis and necrosis pathways. This research employed nsPEFs combined with calcium ions against human sensitive (EPG85-257P) and drug-resistant (EPG85-257RDB) gastric cancer cells. For this purpose, we used relatively low concentrations of calcium to obtain the effect of oxidative stress and to be able to direct cells to apoptotic death. NsPEFs, unlike microsecond electroporation, enable the permeabilization of inner and outer cell membranes; thus, we can deliver drug molecules to all cellular sectors. The observations of nsPEFs effects with calcium ions were evaluated by MTT (3-(4,5-dimethylthiazol-2-yl)-2,5-diphenyltetrazolium bromide) viability assay, flow cytometry, proteasomal activity measurements by luminescent assays, and confocal microscopy. It was observed that nsPEFs can stimulate oxidative and antioxidative effects and apoptosis induction. The obtained results suggest the potential mechanism of nsPEFs action in gastric cancer cells. 

## 2. Results

### 2.1. NsPEFs Viability Effects on Gastric Cell Viability 

**Figure 1:**
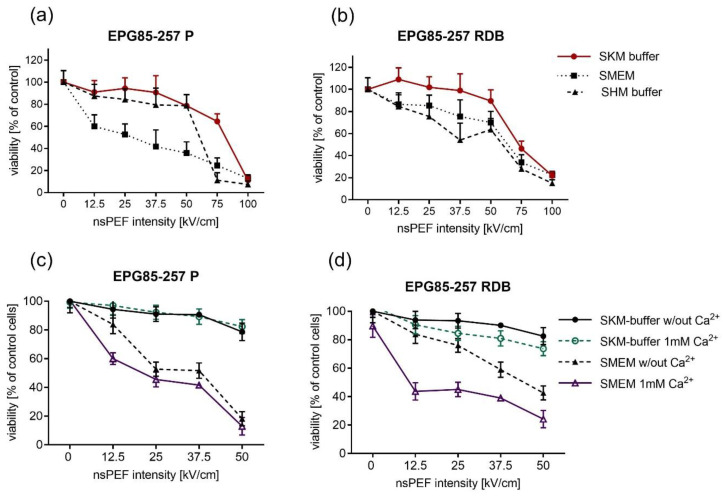
The evaluation of cell viability by MTT assay after 24 h post nsPEF in human gastric cells (**a**) sensitive, and (**b**) drug-resistant; with and without 1 mM calcium ions in (**c**) sensitive, and (**d**) drug-resistant cells. Data are mean ± SD (n = 3). SKM buffer—potassium-based buffer, SMEM—Spinner Modification of Minimum Essential Eagle’s Medium, SHM—10 mM HEPES based buffer.

The effects of nanosecond electric pulses were determined after 24 h by the MTT method. Figure 1 indicates the results from the viability assay without (Figure 1a,b) and with calcium ions (Figure 1c,d). NsPEF exposure was performed in three buffers of varying conductivity. As we can observe, the cytotoxic effect increased simultaneously with the increasing intensity of the electric field. The least cytotoxic occurred SKM buffer (potassium-based buffer) with the lowest conductivity. In the case of sensitive cells, cell viability was reduced the most intensively when the protocol was performed in an SMEM buffer (Spinner Modification of Minimum essential Eagle’s medium). SKM and SHM (HEPES based buffer) buffers caused similar cell viability effects. In the case of drug-resistant cells, SMEM and SHM buffers (HEPES based) caused similar effects on cell viability, SKM buffer occurred less cytotoxic. For 1 mM, calcium ions delivery SKM and SMEM buffers were used (Figure 1c,d). As we can observe in sensitive cells, nsPEF with or without calcium ions caused almost the same cell viability decrease. SMEM with Ca^2+^ induced the highest cytotoxic effect. However, in resistant cells we observed a notable decrease in cell viability after nsPEF with Ca^2+^, particularly in the SMEM buffer.

### 2.2. Cell Membrane Permeabilization

**Figure 2:**
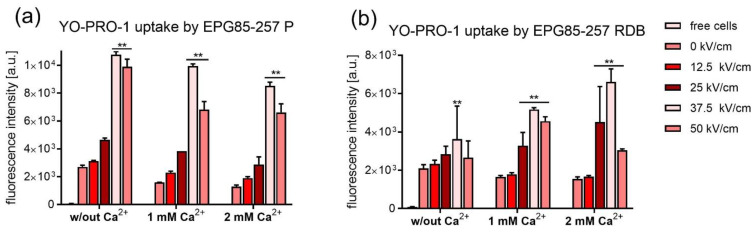
The evaluation of cell membrane permeabilization detected by YO-PRO-1 green dye uptake by (**a**) sensitive, and (**b**) drug-resistant cells. Data are mean ± SD (n = 3 independent experiments and technical repetitions), ** *p* ≤ 0.05.

**Figure 3:**
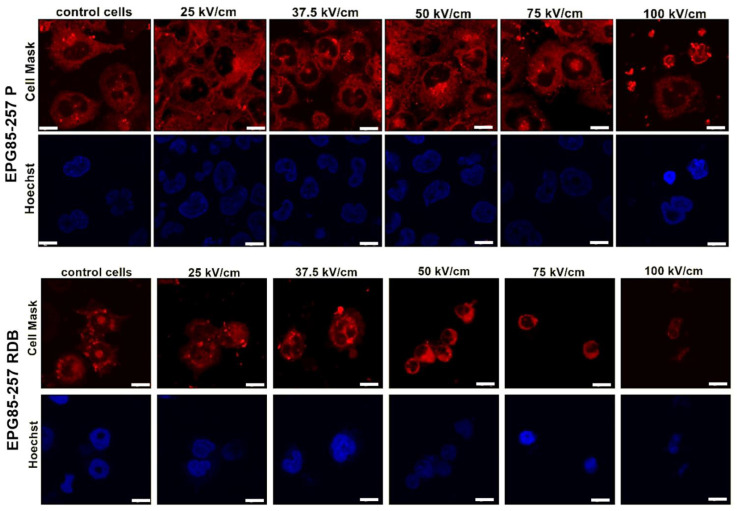
The evaluation of nsPEFs impact on the cell membrane morphology in EPG85-257 P and RDB cells by CLSM imaging, nuclei stained in blue color with DAPI, and cell membranes stained with CellMask Deep Red plasma marker. Scale bars correspond to 10 µm.

The cell membrane permeabilization was determined by impermeable dye Yo-Pro-1 using flow cytometry (Figure 2) and by a fluorescent marker of lipid plasma—CellMask Deep Red, by confocal microscopy visualization (Figure 3). The obtained results demonstrate that cell membrane permeability increased with the increasing intensity of the electric field. In the case of the addition of calcium ions to sensitive cells, we could observe a very slight decrease, but in drug-resistant cells, cell permeabilization increased in the presence of Ca^2+^. Interestingly, after the exposure to 50 kV/cm, cell permeabilization decreased, particularly in the resistant cells. This can be related to the strong effect on lipid membranes caused by this electric field intensity. 

The morphology of cell membranes exposed to nsPEFs is shown in Figure 3. As we can see, in the most intense electric field the most notable alterations in cellular membranes occurred. We could first swelling (25 kV/cm) and then the loss of cell shape, shrinkage (37.5–50 kV/cm), and dismemberment of cellular structures (75–100 kV/cm). This experiment also confirms the decreased Yo-Pro-1 uptake in the case of 50 kV/cm if we consider the poor condition of cell membranes.

### 2.3. F-actin, Fluo-4 and Lipid Droplets Imaging by CLSM Study

**Figure 4:**
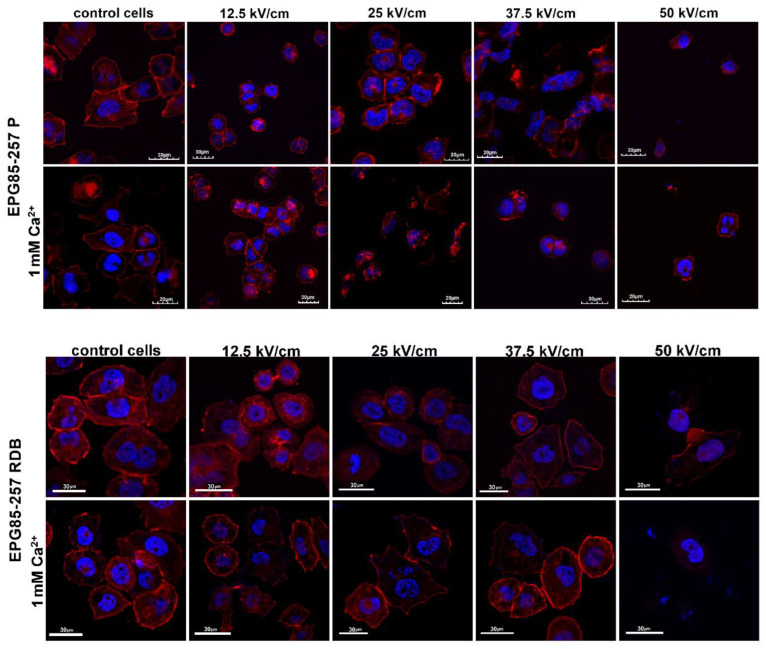
The evaluation of cytoskeleton protein, F-actin (**red**), and nuclei (**blue**) after 24 h in human gastric cells, after the exposure to nsPEFs with or without calcium ions (1 mM), Note: F-actin-red; nuclei—blue.

Confocal microscopy was implemented for the analysis of the cellular cytoskeleton, intracellular calcium, and neutral lipid storage. The visualization of F-actin after nsPEF with or w/o Ca^2+^ post 24 h is shown in Figure 4. In both cell lines, the increasing electric field intensity provoked the reorganization of actin fibers to concentrate on the edge of cells. NsPEFs were more destructive to the cytoskeleton of sensitive cells than drug-resistant ones. In EPG85-257 P cells, evident F-actin reorganization was seen. NsPEF with calcium ions dramatically affected actin fibers and reduced cell numbers. In the case of EPG85-257 RDB cells, exposure to ns pulses caused F-actin to form lamellipodia and taut fibers forming arcs on the edge of the cells. Actin fibers become taut, and their loss from inside the cells is observed, which is confirmed by the intense red fluorescent signal. The combination with 1 mM Ca^2+^ and 50 kV/cm significantly reduced F-actin cytoskeleton. 

**Figure 5:**
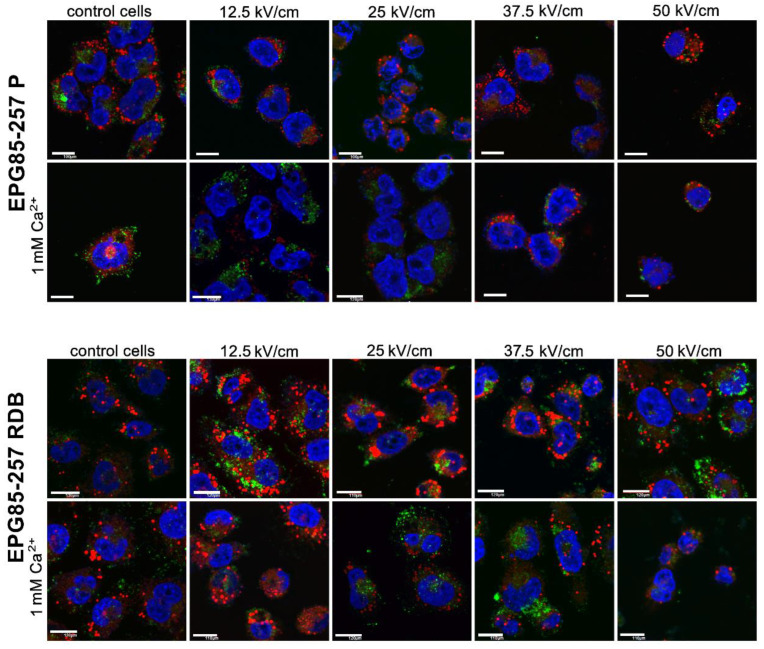
The evaluation of calcium by Fluo-8 green dye and lipid droplets stained by red dye LipidTOX™ Deep Red Neutral Lipid Stain, after 24 h in human gastric cells, after the exposure to nsPEFs with or without calcium ions (1 mM). Nuclei stained by DAPI blue marker.

In the next step, lipid droplets (LDs) and calcium storage in gastric cancer cells post nsPEFs were stained by fluorescent markers. The results are demonstrated in Figure 5. It was noted that in EPG85-257 P cells the exposure to nsPEFs did not change LDs number and distribution. The combination of nsPEFs with calcium ions noticeably reduced the fluorescent signal derived from LDs after 12.5 and 25 kV/cm. In the case of EPG85-257 RDB, after nsPEF increased LDs number and signal intensity, which can be explained as a response to the induced stimuli. A decreased number and distribution of LDs was observed for the parameters 25, 37.5 and 50 kV/cm and the addition of 1 mM Ca^2+^. Fluo-8 staining post nsPEF in sensitive cells resulted in a decreasing green fluorescent signal which corresponds to the calcium release from cells as an effect of cell permeabilization. The addition of 1 mM Ca^2+^ intensified the Fluo-8 signal for 12.5 and 25 kV/cm parameters. For higher nsPEFs parameters, cells were no longer coping and the fluorescent signal decreased. For EPG85-257 RDB cells nsPEFs alone, Fluo-8 signal distribution increased, and the addition of calcium ions resulted in the increased signal for 25 and 37.5 kV/cm parameters. 

### 2.4. Antioxidative and Oxidative Stimulation Effects of nsPEF

**Figure 6:**
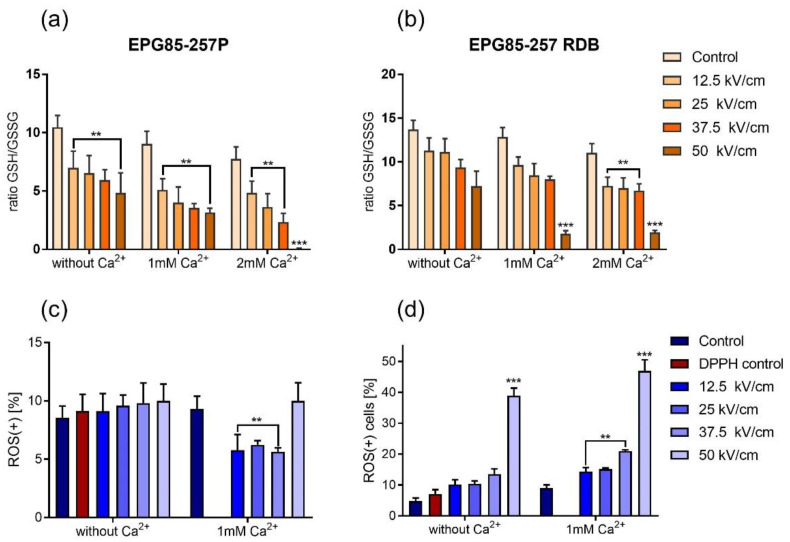
The evaluation of antioxidative and oxidative effects after the exposure of EPG85-257 P and RDB cells to nsPEFs alone or with calcium ions. The ratio of GSH/GSSG in (**a**) sensitive and (**b**) drug-resistant cells. Fluorescent signal detection of reactive oxygen species (ROS) in (**c**) sensitive and (**d**) drug-resistant cells. Data are mean ± SD (n = 3 independent experiments), ** *p* < 0.05, *** *p* < 0.005.

The antioxidative response to nsPEFs with calcium ions was measured after 24 h by the ratio of reduced to oxidized glutathione (GSH/GSSG) (Figure 6a,b). In optimal conditions, this ratio is relatively high in cells [26], and oxidative stress or unfavorable conditions might significantly decrease this marker. In turn, oxidative effects were detected after 60 min post nsPEF by reactive oxygen species (ROS) in human gastric cells (Figure 6c,d). It was observed that for both cell lines the ratio of GSH/GSSG decreased with the increasing intensity of the electric field. The addition of calcium ions (1 or 2 mM) slightly intensified this effect. This indicates the growing damage of thiols and thus increasing protein damage. In the case of ROS release, we observed marginally increasing ROS levels with the increasing electric field parameters in sensitive cells. The addition of calcium ions decreased this effect. In resistant cells, ROS release also rises with the increasing parameters of the electric field and in the presence of Ca^2+^. The most oxidizing parameter here was 50 kV/cm, which was also four-fold higher than in sensitive cells. 

### 2.5. Proteasomal Activity Changes after nsPEF with Calcium Ions

**Figure 7:**
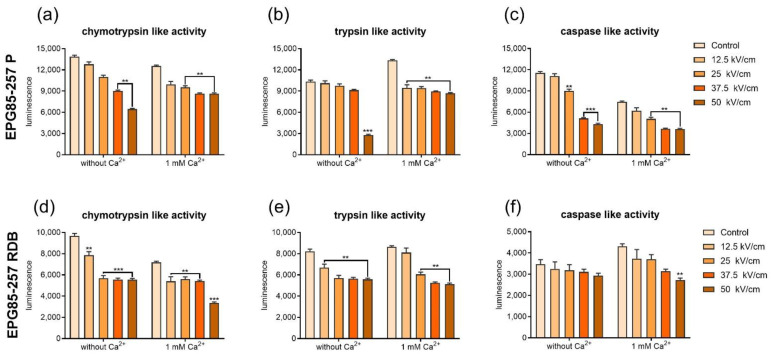
The evaluation of proteasomal activity after the exposure to nsPEFs alone or with calcium ions for EPG85-257 P cells: (**a**) chymotrypsin-like activity, (**b**) trypsin-like activity, (**c**) caspase-like activity, and EPG85-257 RDB cells: (**d**) chymotrypsin-like activity, (**e**) trypsin-like activity, (**f**) caspase-like activity. Data are mean ± SD (n = 3 independent experiments), ** *p* < 0.05, *** *p* < 0.005.

For the proteasomal activity, an ultrasensitive luminescent assay was used, which enabled the detection of three proteasome activities: chymotrypsin-like, trypsin-like, and caspase-like. The obtained results are shown in Figure 7. In the case of both gastric cell lines, a decreasing tendency of all activities was observed, but the addition of calcium ions did not strongly support this effect. 37.5 and 50 kV/cm were the parameter that most affected proteasome activities. 

### 2.6. Apoptosis Induction post nsPEF with Calcium Ions

**Figure 8:**
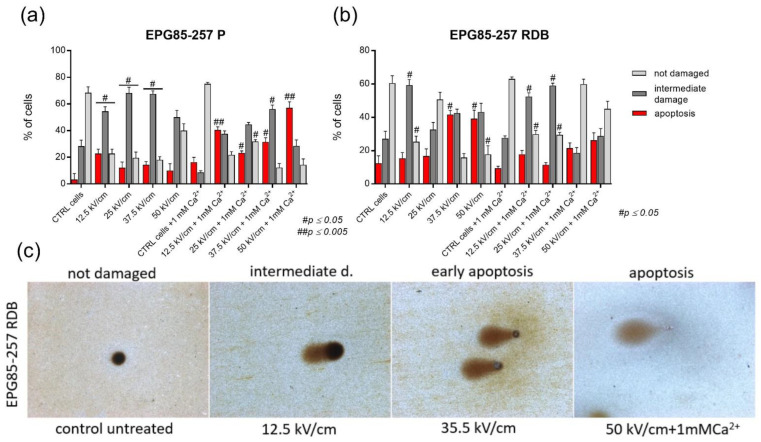
Apoptosis analysis by comet assay in human gastric cancer cells (**a**) P-sensitive, and (**b**) RDB—resistant) after nsPEF protocols, (**c**) exemplary microphotographs of evaluated comets. Magnification 600×.

**Figure 9:**
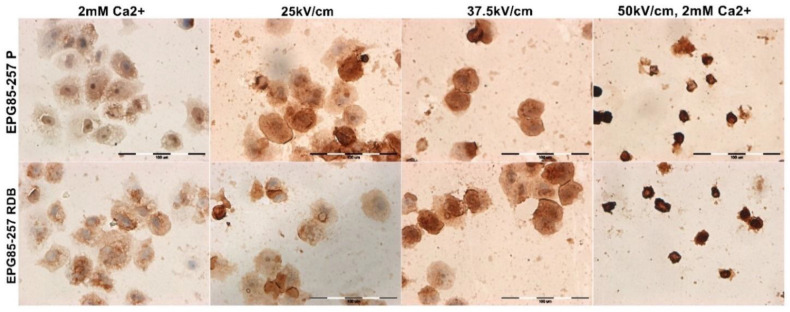
The visualization of AIF protein expression in human gastric cancer cells (P-sensitive, and RDB—resistant) after nsPEF protocols; exemplary microphotographs are shown. Scale bars correspond to 100 µm.

Apoptosis induction post 24 h, after the exposure to nsPEF, was evaluated by comet assay (Figure 8) and AIF (apoptosis induced factor) marker (Figure 9 and Table 1). Figure 8c demonstrated exemplary comets that were judged in the experiment. In sensitive cells, the most pro-apoptotic parameters were 12.5, 37.5 and 50 kV/cm with calcium ions. Interestingly, cells exposed to nsPEFs alone demonstrated a higher number of cells with intermediate damage. In drug-resistant cells, the highest number of apoptotic cells occurred when used at 37.5 or 50 kV/cm. The addition of calcium ions resulted in a higher number of cells with intermediate damage. These cells, defined as “intermediate damage”, can undergo apoptotic or necrotic pathways.

In the next step, apoptosis-inducing factor (AIF) was evaluated by immunocytochemical assay. This oxidoreductase marker plays a role in the induction of the caspase-related apoptosis pathway, and its lack is responsible for the weaker mitochondrial condition [27]. Both controls of gastric cancer cell lines demonstrate some initial level of the expression of AIF. The exposition to nsPEF intensifies the immunostained reaction; after 50 kV/cm the intensive reaction was detected in the nuclear envelope. The addition of calcium ions also intensified the stained reaction in the nuclear envelope and cytoplasm. For the highest parameters, the reduced number of cells was detected in the field of observation. 

## 3. Discussion

Nanosecond electric pulses have been used in biology and industry in recent years, most markedly for small molecule delivery, food processing, and thermal or non-thermal ablation [11,28]. In this study, we proposed the application of ultrashort 10 ns electric pulses with high voltage in combination with a low concentration of calcium ions for the induction of apoptosis and oxidative changes in gastric cancer cells. NsPEF with high intensities caused a decrease in cell viability, but low calcium ion concentration (1 mM) was safe and did not affect cells’ viability. Only the combination of nsPEF and Ca^2+^ caused synergistic action triggered by the cumulative effect of these two factors. Our results demonstrated that the applied nsPEFs protocols induce ROS release, decrease the reduced to oxidized glutathione ratio, affect F-actin fibers, and induce apoptosis. Our study proved that nsPEFs caused F-actin reorganization, in particular in the presence of calcium ions. It was manifested by damaged actin fibers and significantly reduced cell numbers. Interestingly, in resistant cells, nsPEFs caused F-actin to form lamellipodia and taut fibers forming arcs on the edge of the cells. Similarly, Cemazar et al. indicated that electroporation causes depolymerization of microtubules and the depolymerization of actin filaments. Additionally, alterations in cell junctions were also observed [29]. Stacey et al. also proved that nsPEFs sensitized the actin cytoskeleton and induced its depolymerization. It was also noted that nsPEFs affected the nuclear membrane and chromosomal telomere [30]. In another study, the effects of electroporation with cisplatin were determined. The authors performed advanced proteomic analysis and found downregulated proteins, which also included those involved in the regulation of actin cytoskeleton and proteasomes [31]. Interestingly in our study, the proteasomal activity decreased post nsPEF exposure in gastric cancer cells. As we know, proteasomes are the essential sites responsible for protein degradation. Proteasome particles include chymotrypsin-like, trypsin-like, and caspase-like proteolytic sites. These structures are engaged in degrading protein substrates that regulate the cell cycle and prosurvival pathways. The 26S proteasome is also very sensitive to oxidative stress; in particular, sensitive to hydroxyl peroxide, which can affect its activity [32]. The available data show that proteasome inhibition results in the arrest of cell proliferation and apoptosis induction. Thus, in the case of our results, the inhibition of proteasomal activity seems beneficial in gastric cancer therapy. Additionally, some studies indicate that proteasome inhibitors reduce membrane transporters from the ABC family [33], which seems promising in drug-resistant types of cancer. That is why proteasome inhibition is one of the directions of anticancer therapies. In our study, the decrease of three proteasomal activities was also observed in drug-resistant cells. This effect was more potent in the presence of calcium ions. Our previous study also demonstrated a promising anticancer effect of nsPEFs in drug-resistant cells [17], also with calcium ions [34]. In the other studies it was demonstrated that nanosecond electroporation is also effective in drug-resistant bacteria [35,36]. Currently, there is a limited number of papers focusing on the effects of electroporation, in particular in the nanosecond range, on the multi-drug drug-resistance (MDR) phenomenon. MDR is still an unsolved problem in chemotherapeutic protocols, and some types of cancer reveal a high expression of MDR proteins originally, in particular those derived from the digestive system [37]. Multidrug resistance is a multifactorial and complex phenomenon. As can be seen, the most significant difference between sensitive and resistant cells lies in the structure of their cell membranes. The lipid membranes of resistant cells are more packed and decorated with proteins responsible for drug resistance. In response to the MDR phenomenon, PEF is proposed, which is a purely physical method that disintegrates cellular structures. Thus, the rigid lipid barrier loaded with MDR proteins will not be an obstacle in the transport of the drug’s molecules using PEFs. It has been found that resistant cells have a higher impedance modulus and more negative phase than sensitive cells. Moreover, resistant cells revealed a higher cell membrane capacitance and diminished intracellular resistance [38]. Here we demonstrated that ultrashort 10 ns electric pulses could be used as an effective tool against gastric cancer cells with drug resistance. The obtained data suggest that nsPEFs, in particular with calcium ions, initiate apoptotic cell death accompanied by proteasomal activity inhibition, ROS release, and protein and cytoskeleton damage. Thus this method could be considered in future in vivo applications and clinical trials. 

## 4. Materials and Methods

### 4.1. Cell Lines

Two gastric adenocarcinoma cell lines were used: the EPG85-257 P – sensitive and EPG85-257 RDB – daunorubicin-resistant cell line. Human gastric cancer cells were obtained as a kind gift from Prof. Herman Lage. The EPG85-257 RDB cell line reveals the overexpression of MDR1 and the resistance to daunorubicin [37]. Both cell lines were cultured in Leibovitz L-15 medium (Sigma-Aldrich, Poznan, Poland) supplemented by 10% fetal bovine serum (FBS, Lonza, Poland), 1% antibiotics (penicillin/streptomycin, Sigma-Aldrich, Poznan, Poland), 1 mM ultraglutamine (Sigma-Aldrich, Poznan, Poland), 6.25 mg/L fetuin (Sigma-Aldrich, Poznan, Poland), 2.5 mg/mL transferrin (Sigma-Aldrich, Poznan, Poland), 0.5 g/L glucose, 1.1 g/L NaHCO_3_, and 1% minimal essential vitamins (Mem-Vit, Sigma-Aldrich, Poznan, Poland). Cell cultures were cultured as a monolayer on a 25 and 75 cm^2^ plastic flask (Sarstedt, Germany) maintained in a humidified atmosphere at 37 °C and 5% CO_2_, and detached for the experiments by trypsinization (trypsin 0.025% and EDTA 0.02% solution, Sigma-Aldrich, Poznan, Poland). Cells were passed every 2–3 days and a day before the experiment.

### 4.2. Nanosecond Pulses Exposure Protocol with Calcium Chloride

**Scheme 1:**
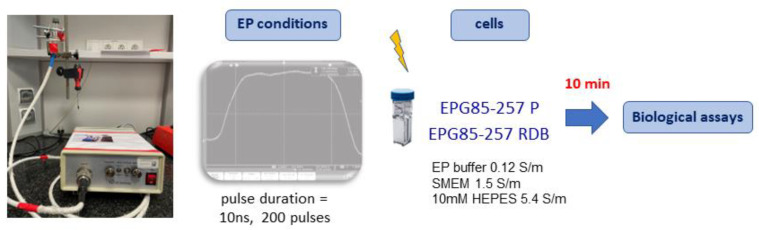
Schematic representation of the experimental setup including the pulse-generation system and the signal shape from the oscilloscope.

Nanosecond pulses (nsPEF) were generated by a PPG-20 generator (FID Technology, Burbach, Germany) and measured by oscilloscope TDS3054 (DPO, 500 MHz, 5 GS/S, 4 channels, Tektronix, Tespol, Poland) with a high-voltage probe (75 MHz, 40 kV, 1000× 10 FT; P6015A, Tektronix, Tespol, Poland). The following electrical field parameters were used: electric field intensity — nsPEF = 12.5–50 kV/cm, repetition frequency—100 Hz, number of pulses—200, pulse duration 10 ns with time rise of 2 ns (Scheme 1).

For viability experiments, cells were suspended in an electroporation buffer (SKM buffer) with low electrical conductivity of 0.12 S/m (10 mM KH_2_PO_4_/K_2_HPO_4_, 1 mM MgCl_2_, 250 mM sucrose, pH 7.4), SMEM (Sigma-Aldrich, conductivity 1.5 S/m) or SHM buffer (10 mM HEPES based, conductivity 0.1 S/m) [34,39]. Cells were then exposed to electric pulses in a 4 mm cuvette with two aluminum plate electrodes (VWR International, Gdansk, Poland). After electroporation, the cells in cuvettes were left for 10 min at 37 °C, centrifuged 5 min at 500× *g*, and resuspended in a cell culture medium for further analysis. In the case of calcium ions delivery by nsPEF, calcium chloride (CaCl_2_, Sigma-Aldrich, Poznan, Poland) was used in the final 1 mM concentration. Calcium chloride solution was prepared in the same buffer as for nsPEF alone. Control cells were treated the same way as cells exposed to all protocols, i.e., warming 10 min at 37 °C and centrifugation; the same culture dishes were used (cuvettes, centrifugation tubes).

### 4.3. Cell Viability by MTT Assay

The cell viability was performed using the MTT cell proliferation assay (Sigma Aldrich, Poznan, Poland). Firstly, gastric cells were subjected to nsPEF alone or with calcium ions in the final concentration 1 mM. After that, cells were incubated 10 min at 37 °C and 5% CO_2_ for membrane resealing. Cells were then seeded on 96-well plates (Sarstedt, Germany) at a density of 3 × 10^4^ cells. Viability was then performed after 24 h according to the manufacturer’s protocol. The absorbance was measured at the wavelength of 570 nm using a microplate reader based on the fluorescent filters (Glomax^®^ Discover, Promega, GmbH, Walldorf, Germany). The cell viability was expressed as a percentage of control untreated cells. All experiments were performed in triplicate.

### 4.4. The Analysis of Cell Membrane Permeabilization by YoPro-1 Uptake and Cell Membrane Staining

Cell membrane permeabilization was detected by the impermeant fluorescent green dye YO-PRO™-1 iodide (YP-1, λ_exc_491/λ_em_509, Thermo Scientific, Warsaw, Poland). YP was added to each sample before electroporation at 1 μM concentration. nsPEF protocols were then performed. Additionally, unstained and stained negative as well as positive controls were included in the experiments. Flow cytometry analysis was performed using FACS Calibur flow cytometer (Becton Dickinson, Franklin Lakes, NJ, USA). The fluorescence of Yo-Pro-1 was excited with a 488 nm laser and assessed with the FL-1 detector. Data was collected by BD FACStation software and analyzed in GraphPad Prism 8 (La Jolla, CA, USA).

Confocal microscopy was used for the study in the case of membrane staining and visualization. For the experiment, cells were seeded on cover microscopy slides (24 × 24 mm, Carl Roth GmbH, Karlsruhe, Germany) placed in 35 mm Petri dishes (Nunc, Biokom, Janki, Poland), and left overnight. Then, the cell medium was replaced by an electroporation buffer, and a two needle steel electrode (9 mm gap, custom made by FID Technology, Germany [6]) was used for electric pulse delivery. Cells were then stained with 200 nM CellMaskDeepRed^®^ (Thermo Scientific, Warsaw, Poland). The imaging was performed by 635 nm excitation wavelength and 693 nm emission wavelength. The imaging was performed on the Olympus FluoView FV1000 confocal laser scanning microscope (Olympus, Tokyo, Japan).

### 4.5. F-actin, Fluo-4, and Lipid Droplets Imaging by CLSM Study

Cells were trypsinized and seeded on microscopic cover glasses (24 × 24 mm, Carl Roth GmbH, Germany) in 35 mm Petri dishes (Nunc, Biokom, Janki, Poland). Cells were left to adhere overnight. Cells were then exposed to nsPEF or nsPEF with 1 mM Ca^2+^ or 2 mM Ca^2+^, and left for 24 h. After this time, cells were fixed 10 min in 4% paraformaldehyde (Polysciences, Inc., Bergstrasse, Germany) and washed in PBS (BioShop, EPRO, Poland). Cytoskeleton reorganization was determined by immunofluorescent cells’ labeling of F-actin with Alexa Fluor^®^546 Phalloidin (Thermo Fisher, distributor: Life Technologies, Warsaw, Poland), and intracellular calcium was determined by 4 µM Fluo-4 (λ_Exc._494/λ_Em._506 nm, F14201, Thermo Fisher Scientific), lipid droplets by HCS LipidTOX™ Deep Red Neutral Lipid Stain (H34477, Thermo Fisher Scientific). FluorshieldTM with DAPI (4,6-diamidino-2-phenylindole) was used to visualize the nuclei and to mount the cells, and was excited by a 405 nm-excitation channel. The samples were studied on the Olympus FluoView FV1000 confocal laser scanning microscope (Olympus, Tokyo, Japan).

### 4.6. ROS Release Measurements

For the ROS production after nsPEF and nsPEF combined with calcium ions, the DCF method was used. Cells were exposed to nsPEF protocols and resuspended on black 96-well plates with a flat transparent bottom (Perkin Elmer, Krakow, Poland). DCF (2,7-dichlorofluorescein) assay (Life Technologies, Warsaw, Poland) for ROS detection was implemented after 60 min post-electroporation. This method is based on the application of fluorescent properties of 6-carboxy-2,7-dichlorodihydrofluorescein diacetate 2,7-dichlorofluorescein (carboxy-H_2_DCFDA). The stock solution of carboxy-H_2_DCFDA (50 µg/mL in sterile DMSO) was kept at RT in dark conditions and was afterward diluted, following the manufacturer’s protocol, in a cell culture medium without FBS. As a positive ROS control, an oxidizing agent 2, 2-diphenyl-1-picrylhydrazyl (DPPH, Sigma-Aldrich) in 100 µM concentration was used. Then, the DCF reagent was added to the cell culture to achieve a final concentration of 10 µM and was left in darkness for 30 min of incubation at 37 °C. After this time, the fluorescence was measured, where the excitation wavelength of 495 nm and emission wavelength of 530 nm was used. The ROS level was detected by a multiwell scanning spectrophotometer (EnSpire Perkin Elmer, Warsaw, Poland).

### 4.7. The Level of Reduced and Oxidized Glutathione (GSH/GSSG)

After nsPEFs exposure with or without calcium ions, cells were seeded into white 96-well microculture plates at the concentration of 5 × 10^3^ cells/well. The level of reduced and oxidized glutathione was determined after 24 h by a luminescence-based assay (GSH/GSSG-Glo™ Assay, Promega, Walldorf, Germany) using a multiwell scanning spectrophotometer at 570 nm (EnSpire Perkin Elmer, Warsaw, Poland). The experimental procedure was performed according to our previous study [40]. Experiments were repeated in triplicate. The results were expressed as a ratio of the reduced and oxidized glutathione (GSH/GSSG).

### 4.8. Proteasomal Activity

The activity of chymotrypsin-, trypsin- and caspase-like proteases (Cell-Based Proteasome-Glo™ Promega, Walldorf, Germany) associated with the proteasome complex in cultured cells was determined after 24 h in cells after PEFs. This activity is responsible for much of the protein degradation required to maintain cellular homeostasis, including the degradation of critical cell-cycle proteins, tumor suppressors, transcription factors, inhibitory proteins, and damaged cellular proteins [41]. Cells after nsPEFs protocols with or w/o calcium ions were seeded into white 96-well microculture plates at the concentration of 5 × 10^3^ cells/well. The activity of three proteasomal activities were determined after 24 h by a luminescence-based assay using a multiwell and multimode scanning spectrophotometer (EnSpire Perkin Elmer, Poland).

### 4.9. Cell Death Evaluation by Neutral Comet Assay

For the evaluation of DNA fragmentation associated with apoptosis, the neutral comet assay (NCA)—a method described by Collins—was used [42]. The cells at a concentration of 1 × 10^5^/^mL^ were mixed with a low-melting agarose at a ratio of 1 to 10 volumes (*v*/*v*) and was then spread on basic microscope slides. The slides were submerged in a precooled lysis solution (2.5 M NaCl, 100 mM EDTA, pH 10, 10 mM Tris base and 1% Triton X-100) at 4 °C for 60 min. After rinsing, the slides were equilibrated in TBE solution for neutral conditions (40 mM Tris/boric acid, 2 mM EDTA, pH 8.3), electrophoresed at 1.0 V/cm^2^ for 20 min, and then silver staining was performed. For scoring the comet pattern, 100–200 nuclei were counted from each slide. To rank apoptotic comets, we followed the method developed by Collins [42].

### 4.10. Immunocytochemical Analysis of Apoptosis Inducing Factor (AIF)

Immunocytochemical ABC reaction was performed to investigate the effect of nsPEF with or without 1 mM or 2 mM Ca^2+^ on the expression of apoptosis-inducing factor (AIF) in gastric cancer cells. After the exposure to nsPEF protocols, the cells were seeded on 8-well diagnostic microscopic slides (Thermo Fisher Scientific, Warsaw, Poland), and incubated for 24 h. The cells were then fixed using 4% paraformaldehyde (PFA, Sigma-Aldrich) for 10 min. For the immunocytochemical assay, slides were stained using the EXPOSE Mouse and Rabbit Specific HRP/DAB Detection IHC kit (Abcam, Cambridge, UK,; Cat. no. ab80436). The enzyme expression was visualized with the mouse monoclonal antibody anti-AIF (E-1) (Santa Cruz Biotechnology, Inc,, Dallas, Texas, USA, Cat. no. sc-13116) diluted 1:200 with the antibody diluent (Merck, Millipore, Poznan, Poland). After 1 h incubation at 37 °C with the primary antibody, the cells were incubated with the secondary antibody conjugated with horseradish peroxidase (HRP). Next, samples were incubated with a diaminobenzidine–H_2_O_2_ mixture to visualize the HRP label. Between the steps, samples were rinsed using 1% Triton X-100 in PBS (IITD, Wroclaw, Poland). The cells were stained with hematoxylin (Carl Roth GmbH, Karlsruhe, Germany) for 3 min to visualize the nuclei. The immunocytochemically stained reaction was evaluated with a double-blind method using an upright microscope (Olympus BX51, Tokyo, Japan). The results were judged to be positive if staining was observed in more than 5% of cells. The intensity of immunohistochemical staining was evaluated as (–) negative (no reaction), (+) weak, (++) moderate, and (+++) strong. All experiments were repeated three times.

### 4.11. Statistical Analysis

The statistical analysis was performed using GraphPad Prism 8 (La Jolla, CA, USA). The results were analyzed by two-way ANOVA. Results were expressed as mean ± standard deviation (SD) of the mean and where *p* < 0.05 was considered statistically significant.

## 5. Conclusions

Physical methods are the purest tools for cell manipulation and can be efficiently utilized in support of anticancer protocols. Although it has promising effects, the PEFs technique using nanosecond pulses is still not used in clinical practice. Recent studies show that calcium ions are an effective anticancer agent and are used in electrochemotherapy. However, the list of cancers qualified for this treatment remains too short. In this study, we used low calcium ions as an anticancer agent combined with ultrashort electric pulses (10 ns) of high voltage, which is a proposition of a safe and relatively economical approach. The results presented in this study reveal that the nsPEF method affects gastric cancer cells’ metabolism and induces apoptotic cell death, particularly with calcium ions. Additionally, nsPEF modality provokes membrane permeabilization, oxidative stress, and protein degradation; thus, this method can be a suitable choice for current anticancer pharmacological methods, in particular with drug resistance. So far, electroporation-based methods are not clinically approved for gastric cancers. Thus, there is a need to conduct research in this direction, so each result brings us closer to developing medical protocols.

## Figures and Tables

**Table 1:** Immunocytochemical evaluation of AIF (apoptosis inducing factor) in gastric cancer cells after nsPEFs therapy with calcium ions.

Protocol	EPG85-257P	EPG85-257RDB
control cells	100% + (cytoplasm)	100% ++ (cytoplasm)
12.5 kV/cm	100% ++	100% ++/+++
25 kV/cm	100% ++	100%, nuclear envelope +; cytoplasm ++
37.5 kV/cm	100% ++	100% ++/+++
50 kV/cm	100% ++ (nuclear envelope)	100% ++/+++
1 mM Ca^2+^	100% +	100% ++
12.5 kV/cm + 1 mM Ca^2+^	100% ++	100% ++/+++
25 kV/cm + 1 mM Ca^2+^	100% ++/+++ (nuclear loc.)	100% ++/+++ (nuclear loc.)
37.5 kV/cm + 1 mM Ca^2+^	100% +++ nuclear envelope localization	98% ++/+++ Nuclear envelope localization
50 kV/cm + 1 mM Ca^2+^	100% +++ reduced cell number	100% +++ reduced cell number
2 mM Ca^2+^	100% +	100% +/++
12.5 kV/cm + 2 mM Ca^2+^	100% +	100% +/++
25 kV/cm + 2 mM Ca^2+^	100% ++ (nuclear envelope)	100% ++/+++ (nuclear envelope)
37.5 kV/cm + 2 mM Ca^2+^	100% ++ (nuclear and cytoplasm)	100% ++/+++ (nuclear and cytoplasm)
50 kV/cm + 2 mM Ca^2+^	100% +++ damaged cells (nuclei)	100% +++ damaged cells (nuclear localization)

The immunohistochemical staining was evaluated as (+) weak, (++) moderate, and (+++) strong.

## Data Availability

Not applicable.
